# TEM Nanostructural Investigation of Ag-Conductive Filaments in Polycrystalline ZnO-Based Resistive Switching Devices

**DOI:** 10.1021/acsami.0c05038

**Published:** 2020-06-08

**Authors:** Katarzyna Bejtka, Gianluca Milano, Carlo Ricciardi, Candido F. Pirri, Samuele Porro

**Affiliations:** † Center for Sustainable Future Technologies @ POLITO, Istituto Italiano di Tecnologia, Via Livorno 60, Turin 10144, Italy; § Department of Applied Science and Technology, Politecnico di Torino, C.so Duca degli Abruzzi 24, Turin 10129, Italy

**Keywords:** resistive switching, memristor, Ag-conductive filament in ZnO, grain boundaries, TEM

## Abstract

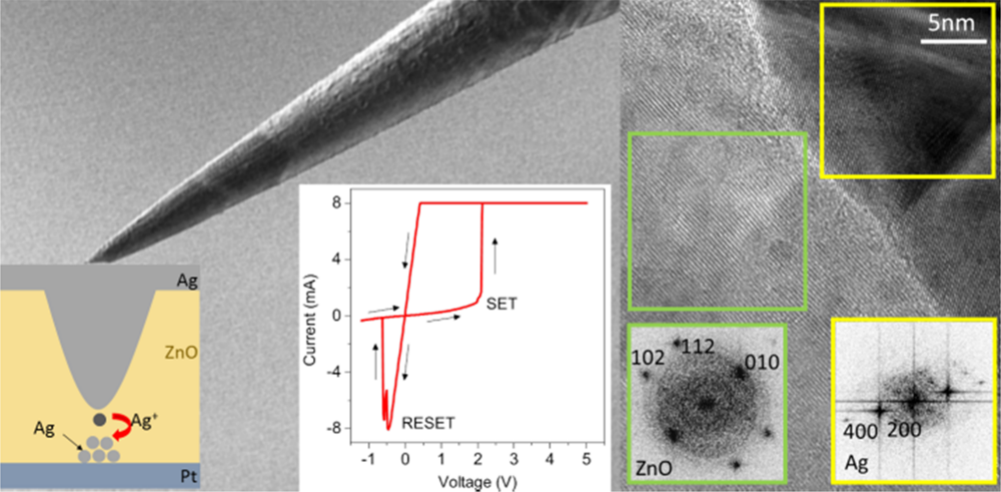

Memristive devices based on a resistive switching mechanism are considered very promising for nonvolatile memory and unconventional computing applications, even though many details of the switching mechanisms are not yet fully understood. Here, we report a nanostructural study by means of high-resolution transmission electron microscopy and spectroscopy techniques of a Ag/ZnO/Pt memristive device. To ease the localization of the filament position for its characterization, we propose to use the guiding effect of regular perturbation arrays obtained by FIB technology to assist the filament formation. HRTEM and EDX were used to identify the composition and crystalline structure of the so-obtained conductive filaments and surrounding regions. It was determined that the conducting paths are composed mainly of monocrystalline Ag, which remains polycrystalline in some circumstances, including the zone where the switching occurs and at secondary filaments created at the grain boundaries of the polycrystalline ZnO matrix. We also observed that the ZnO matrix shows a degraded quality in the switching zone, while it remains unaltered in the rest of the memristive device.

## Introduction

Memristive devices are widely studied to address the global increasing demand for fast, low-power, and scalable memory technologies and the realization of brain-inspired neuromorphic hardware architectures.^1−5^ Two terminal memristive devices are usually realized by sandwiching an insulator matrix in between two metal electrodes by forming a metal–insulator–metal (MIM) structure. Working principles of memristive devices rely on the resistive switching mechanism responsible for the change of the internal resistance state of the device when subjected to electrical stimulation. In redox-based resistive switching devices, this mechanism relies on nanoionic effects responsible for atomic reconfiguration of the device.^6^ When an electrochemically active electrode (such as Ag or Cu) is involved, resistive switching occurs due to the so-called electrochemical metallization memory effect (ECM).^7^ In this case, a positive voltage applied to the electrochemically active electrode is responsible for dissolution and electromigration of metal ions across the insulating layer to form a conductive path bridging the two metal electrodes. The formation/rupture of this conductive path upon electrical stimulation is responsible for the switching between low- and high-resistance states of the device. As an alternative, resistive switching can be obtained by sandwiching an insulator matrix between two electrochemically inert metal electrodes (for example Pt or W), by exploiting the valence change mechanism (VCM) effect where the formation/rupture of a conductive path is attributable to the electromigration of oxygen-related defects in the metal-oxide insulating matrix.^6^ To date, resistive switching was observed in a wide range of materials including metal-oxide thin films,^8−12^ nanostructures,^13−16^ and 2D materials^17−20^ in both the sandwich and planar device structure.

Although the switching mechanisms were widely investigated in recent years, with particular focus on the creation, evolution, and dissolution of the conducting filament, many details are yet to be fully understood. In particular, the microscopic nature of the filament and whether one or more filaments are created during the initial electroforming process remain themes of debate. In addition, the study of the changes within the storage media of the tested device is even more neglected. This is related to the fact that the random location of the switching filament creation in a macroscopic device makes it difficult to characterize. In recent works, in situ biasing TEM studies shed some light on understanding the nucleation and growth processes of conducting filaments. Kwon et al. showed that in Pt/TiO_2_/Pt device switching occurs by the local change of the crystalline phase, namely, the formation and disruption of filaments of the Magnéli phase.^21^ Liu et al. obtained in Ag (or Cu)/ZrO_2_/Pt-based devices a real-time observation of the filament formation and dissolution processes based on a local redox reaction inside the ZrO_2_ electrolyte system.^22^ Here, the filament growth was observed from active to inert electrode during the set process. Similar behavior was observed by Yuan et al. in Cu/SiO_2_/W devices.^23^ Conversely, Hubbard et al. observed that the filament grows backward toward the source metal electrode in Cu/Al_2_O_3_/Pt devices.^24^ The latter work claimed that many drawbacks might arise during this type of measurement because often the devices prepared for in-situ characterization have an unrealistic design presenting exposed interfaces, which are vulnerable to surface migration and environmental effects.^24^ This aspect needs to be taken in consideration, even though the in situ studies have been proved to be a useful tool to improve the understanding of the switching mechanisms.^25−28^ It is important to notice that the structures realized for in situ studies are often not equivalent to those studied ex situ, as one dimension has to be drastically reduced. Therefore, the devices studied in situ are rather planar devices. The dynamics of the filament creation can certainly be observed; however, there remains a question whether the behavior is representative for real devices. Milano et al. showed that resistive switching in nanoscale memristive devices based on ZnO nanowires with asymmetric electrodes made of Ag and Pt is highly localized to the nanowire surface.^15^ In that case, the Ag nanocluster filament was created on the surface, and an evidence of the presence of Ag within the nanowire was not found. This was attributed to the fact that Ag ions showed higher mobility at the surface than within the bulk because of the absence of space constrains. The formation and rupture of the Ag nanoisland chains at the surface of nanowires were also observed by Qi et al. in Na-doped ZnO nanowire memristive devices.^16^ In planar Ag/TiO_2_/Pt memristive devices, the formation and rupture of Ag nanofilament were observed by in situ FESEM, while cross-sectional TEM analysis confirmed that the filament was created by Ag particles laying on the surface.^29^ Taking in consideration the above-mentioned research and the structures discussed, which rarely represent the realistic devices, in situ tomography could be very helpful to evaluate the actual shape and position of the filament.^28^

Another approach, which can facilitate the observation of the filaments in representative devices, can be the application of methods aiming to favor the precise creation of filament growth in a given position, e.g., by the insertion of nanoparticles^30^ and the manipulation of the electric field, which acts as an electric field concentrator.^31−34^ This procedure, in addition to improving the performances of resistive switching devices, facilitates the study of the created filaments, as it is possible to prepare samples for TEM analysis at the specific point where filaments are created. Sun et al. investigated the guiding effect of nanoindentation on the growth of conductive filaments in memristive devices and observed improved resistive switching properties, including larger ON/OFF ratios.^31^ TEM analyses confirmed a reduction of the thickness of the active region at the indentation. You et al. created Ag nanocones as top electrodes on SiO_2_ structures and observed selective and controlled filament growth with enhanced performance.^32^ In both cases, any information about the morphology of the filaments was missing. Shin et al. studied pyramidal Ag/Al_2_O_3_/Pt structures and observed the filament creation at the tip of the pyramids.^33^ In all cases, enhanced properties were observed; however, only in the latter work a morphological characterization of the filament was performed: TEM and EDX have shown a distinct filament at the tip region of the pyramids, which was made of Ag and was connecting the Ag and Pt electrodes. However, also in this case, information about the crystalline structure of either the filament or matrix was missing. Gao et al. applied a chemically active metal cathode to construct cone-shaped conducting filaments, with the aim of reconstructing the filament at the same point after its rupture.^34^ In that way, the switching uniformity was improved and the filaments were observed by TEM and EDX. For the observed filaments, a limited characterization is usually given, which includes imaging often in the STEM mode and EDX mapping. However, the crystal structure is rarely discussed; therefore, there is limited information on the nature of the filaments and on the modification of the matrix during the device cycling. For instance, Yang et al. showed a change of crystallinity of the active region in Ag/ZnO:Mn/Pt devices.^8^ The initial polycrystalline ZnO:Mn structure with weak *c*-axis orientation changed to a more disordered polycrystalline wurtzite structure after the device testing. However, it is not specified whether the observed changes are localized close to the filament, and if the modification is later recovered.

In this work, we prepared memristive devices based on polycrystalline ZnO with asymmetric electrodes made of Ag and Pt in the ECM configuration with the aim of analyzing the nanostructure of the conductive filaments by TEM after resistive switching operations. FIB technology was used to assist the fabrication/creation of regular perturbation arrays by patterning the substrate before the deposition of top electrodes. The ion beam that scans the surface leads to the creation of the desired array of protrusions with various depths, depending on the ion beam parameters. In turns, this changes the film morphology and promotes the concentration of the electric field in a near proximity of to the drilled features. This procedure is expected to influence the electrical characteristics of the resulting memristive devices, decreasing the forming voltage and permitting a TEM characterization of the filaments created in correspondence of the prepared perturbation. An extensive insight into the structural nature of conducting filaments under real operation conditions of the devices is reported. Finally, based on the proposed methodology and measurements, a discussion on the nature of the crystalline structure of Ag at both the filament and surrounding areas (named bridge region in this work) and at ZnO grain boundaries is provided. To understand the resistive switching behavior, the characterization of the switching filament should be coupled with a comprehensive analysis of the structure of the surrounding material composing the storage matrix, which is typically disregarded in the published literature. To address this issue, we also discuss the change in the crystallinity of the ZnO switching material near the Ag-conductive path.

## Experimental Details

### ZnO Synthesis

Commercially available oxidized Si wafers were used as substrates. A bottom electrode consisting of a Ta adhesion layer (∼20 nm) and a Pt electrode layer (∼100 nm) was first deposited by sputtering. After that, a ZnO film was deposited by low-pressure chemical vapor deposition (LP-CVD), using the catalytic properties of Pt without the use of any additional seeding layer. The LP-CVD was performed at 650 °C for 20 min, using 300 sccm of Ar as a carrier gas and 150 sccm of O_2_ as a gas precursor. The pressure was fixed at 1.2 Torr during the process. The synthesis procedure is detailed in previously published works.^9,35,36^

### Device Fabrication

ZnO is used as a switching material, Ag as the top electrode (TE), and Pt as the bottom electrode (BE) for the two-terminal devices studied in this work. To prepare devices with small dimensions, their fabrication was assisted by electron-beam lithography (EBL) and focused ion beam (FIB). The patterning was performed in a Zeiss Auriga dual-beam system, equipped with a Raith (ELPHY Quantum) system, which can control both the ion and electron beam.

Starting from as-grown ZnO structures, an array of protrusions were processed by FIB, to obtain a confinement of the electric field and facilitate the creation of the filament in a precise spot. To produce the protrusions with different depths, the milling was performed at 30 kV with an ion current at 20 pA, whereas ion doses were set to 2, 6, 8, and 10 pC/μm^2^ for doses 1, 2, 3 and 4, respectively, by increasing the milling time.

Finally, Ag top electrodes were deposited by EBL (ELPHY Quantum), lift-off and Ag deposition (70 nm, Kurt J. Lesker, PVD 75). The Ag top electrodes are circular in shape and 2 μm in diameter, defining the lateral geometry of the devices.

### Device Characterization

The morphologies of the grown structures and devices, including the characterization of filaments formed during the electrical measurements, were investigated by field emission scanning electron microscopy (FESEM, Zeiss dual beam Auriga), transmission electron microscopy (TEM), and energy-dispersive X-ray spectroscopy (EDX).

TEM investigation was carried out using a FEI TECNAI F20ST microscope, equipped with a field emission gun (FEG) operating at 200 kV. High-angle annular dark field (HAADF) and energy-dispersive X-ray spectroscopy (EDX with EDAX SUTW Si–Li X-ray detector) detectors were used in the scanning TEM (STEM) mode.

Samples for TEM characterization have been prepared in cross section via a standard lift-out technique, using FIB operated at 30 kV, as described previously.^37^ A final cleaning step using a FIB voltage of 2 kV was also performed. Fast Fourier transform (FFT) calculations were performed by using digital micrograph (GATAN) software.

The *I*–*V* characteristics of the Ag/ZnO/Pt structures were performed in situ in the FESEM-FIB chamber equipped with two Kleindiek manipulators in vacuum conditions (6 × 10^–6^ Torr) at room temperature, using an Agilent multimeter and Keithley 4200 semiconductor parameter analyzer.

## Results and Discussion

### Device Fabrication

**Figure 1:**
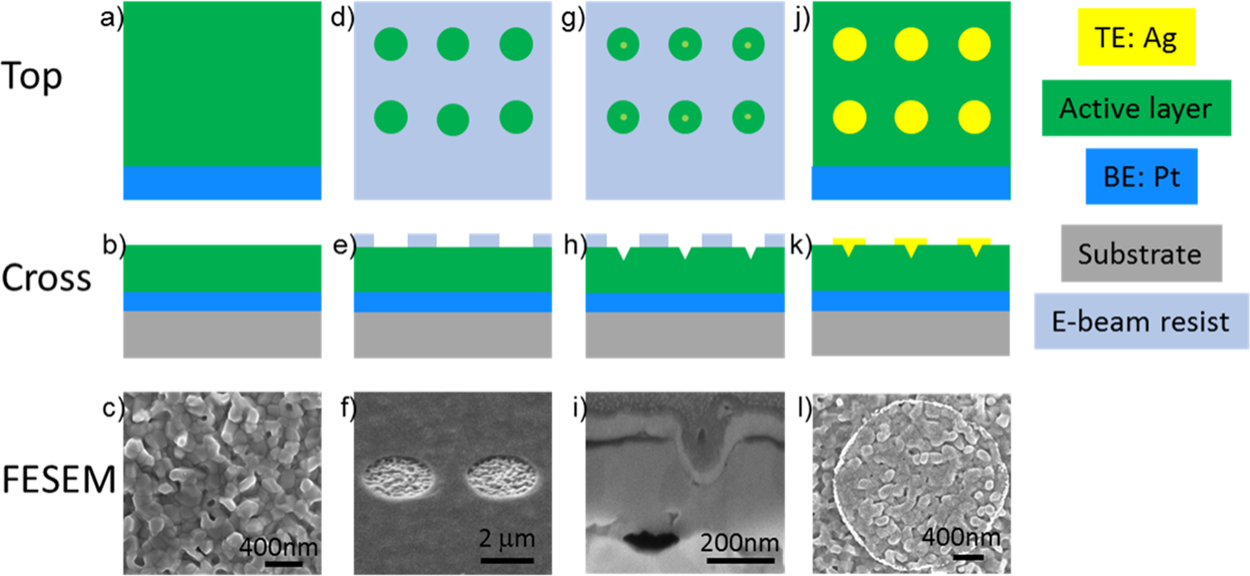
Top and cross-sectional schematics and FESEM images of the fabrication process showing: (a)–(c) as-grown structure, (d)–(f) EBL for definition of the position of TE, (g)–(i) creation of the protrusion via FIB, and (j)–(l) TE deposition and lift out.

Figure 1 depicts the schematic diagram of the device fabrication process and shows a FESEM image in the top or cross-sectional view at each step. Figure 1a–c shows the as-grown ZnO film, which grows with a polycrystalline wurtzite structure with a weak *c*-axis orientation, as characterized in previously published works,^9,35,36^ deposited on top of Pt, which serves as a bottom electrode. In the first step of fabrication, a positive resist was spinned onto the sample. An array of circles with a radius of about 2.5 μm was fabricated after the EBL patterning and development, as shown in Figure 1d–f. The patterned circles define the position where the top electrodes are deposited in the next step and determine the lateral size of a device (∼2.5 μm). FIB patterning is successively aligned so that protrusions with various depth depending on the ion beam parameters are created in the center of the circles, as shown in Figure 1g–i. This procedure changed the morphology of the active layer, resulting in the possibility of concentrating the electric field near the drilled features, which is expected to localize the formation of conductive filaments during resistive switching operations.^31−33,38^Figure 1i shows the FESEM cross-sectional image of one of the protrusions prepared by FIB at a circle’s center. The same image shows the bottom and top electrodes on both sides of the active region. An additional layer is visible at the top of the structure, which corresponds to the Pt protection cap layer deposited during the preparation of the cross section, and it is not part of the device. At last, the top electrode is deposited, followed by the lift-out process, as reported in Figure 1j–l.

The described approach permits to confine the effective dimensions of the devices and localize the filament formation, facilitating the investigation of the obtained filaments by TEM characterization. Figure 1l reports the top view of the final device, including the top electrode. All the preparation steps are carried out at room temperature.

A low-magnification TEM image of the cross section of a device is also shown in Figure S1 a from which it is possible to measure the thicknesses of Ag TE, Pt BE, and the ZnO active layer, which are about 70, 200, and 300 nm, respectively, while the lateral size of a device is ∼2.5 μm (not shown).

Figure S2 shows top-view FESEM images of a sample with multiple devices fabricated on the same substrate, with sufficient space left between the devices to allow the lamella extraction for TEM.

### Resistive Switching Behavior

**Figure 2:**
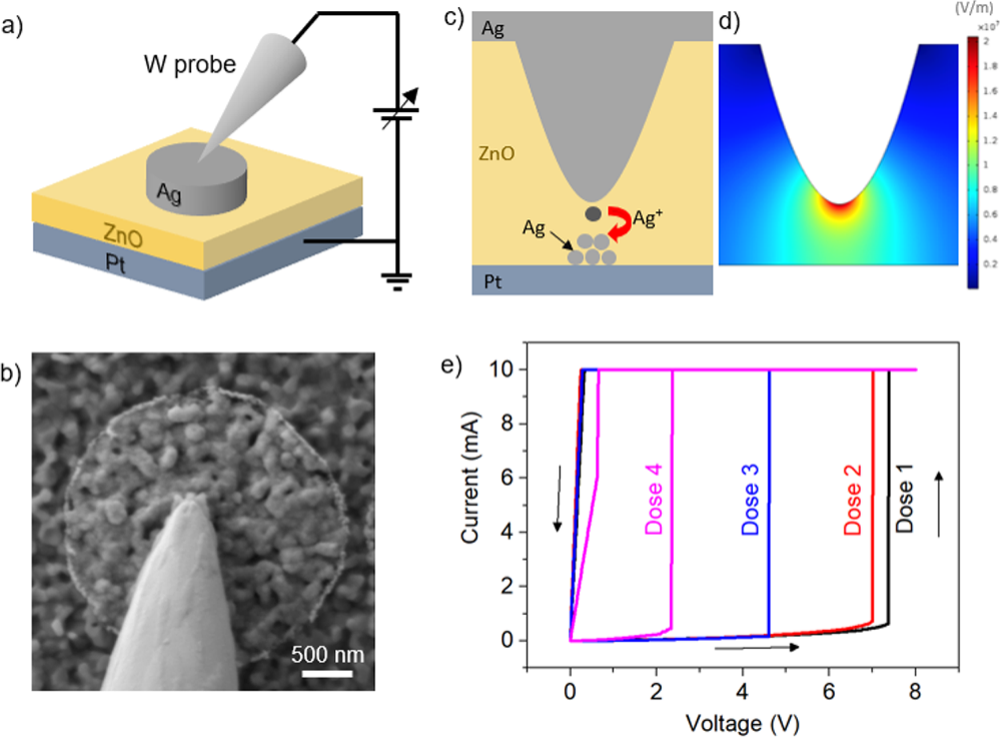
(a) Schematization of the setup for electrical characterization. (b) SEM image of the submicrometric probe contacting the Ag top electrode. (c) Schematization of the resistive switching mechanism involving dissolution of Ag atoms, migration of Ag^+^ ions, and Ag recrystallization to form a conductive path. (d) Simulation of the electric field distribution with enhancement of the electric field at the perturbation tip end; in the simulation, 1 V was applied to the top electrode. (e) Electroforming of devices with perturbations formed by means of different FIB doses where dose 4 > dose 3 > dose 2 > dose 1, as detailed in Experimental Details.

A schematic representation of the device with electrical connections is shown in Figure 2a. The device characterization was performed within the FIB-FESEM chamber with the use of sub-micrometer-sized manipulators. Figure 2b shows a FESEM image of the micromanipulator tip in contact with the top electrode, ready for testing the device. As previously mentioned, asymmetric electrodes of Ag and Pt were considered in this study. Figure 2c reports a schematic representation of the cross section of an Ag perturbation as fabricated by the FIB process, locally reducing the gap from Ag TE toward Pt BE. The mechanism of filament formation by Ag dissolution and Ag^+^ ion migration under electrical bias is also depicted. As can be observed in Figure 2d by finite element method simulations (COMSOL), the FIB-induced indentation is responsible for a local strong enhancement of the electric field. For this reason, dissolution and electromigration of Ag ions occur preferentially in the correspondence of the tip end resulting in a high localization of conductive filament formation. Similarly, strong localization of filament formation due to local enhancement of the electric field was reported in previous works.^32,33,38^

The electrical characterization of the pristine state resistance of Ag/ZnO/Pt devices, performed in a low voltage range for preventing migration of Ag^+^ ions, exhibited a diode-like behavior attributable to the Schottky barriers at the metal/semiconductor interface, as shown in Figure S3. The asymmetric *I–V* characteristic of the pristine state can be attributed to the junction properties with different metal work functions and chemical properties at Ag/ZnO and Pt/ZnO interfaces, as discussed in previous works.^15,39^ To investigate the leakage current of the devices with different thicknesses of the matrix before electroforming, we have investigated the *I–V* characteristic in the pristine state in a small voltage range, as shown in Figure S4a. The Schottky behavior was observed even for the highest dose used for the creation of the perturbation and therefore the thinnest layer of the ZnO switching matrix, and this confirms that the insulator layer is still present. Instead, the electroforming process involving dissolution of metal atoms and migration of Ag^+^ ions across the ZnO matrix is reported in Figure 2e (it is also shown in the semilogarithmic scale in Figure S4b). As can be observed, by increasing the positive applied voltage to the Ag electrode, it is possible to observe a sharp current transition, arising from the formation of an Ag-conductive path in between the electrodes. It is worth noticing that, as expected, a strong dependence of electroforming voltage on the FIB dose used for the creation of the perturbation was observed. Indeed, the voltage needed for the initial creation of the conducting filament exhibited a severe reduction with the increasing dose. As previously discussed, this is due to the reduced thickness at the point where the perturbation is created, together with the associated increase of the electric field that influences the first transition (the so-called electroforming process) of the device from the pristine state to the low-resistance state (LRS).

**Figure 3:**
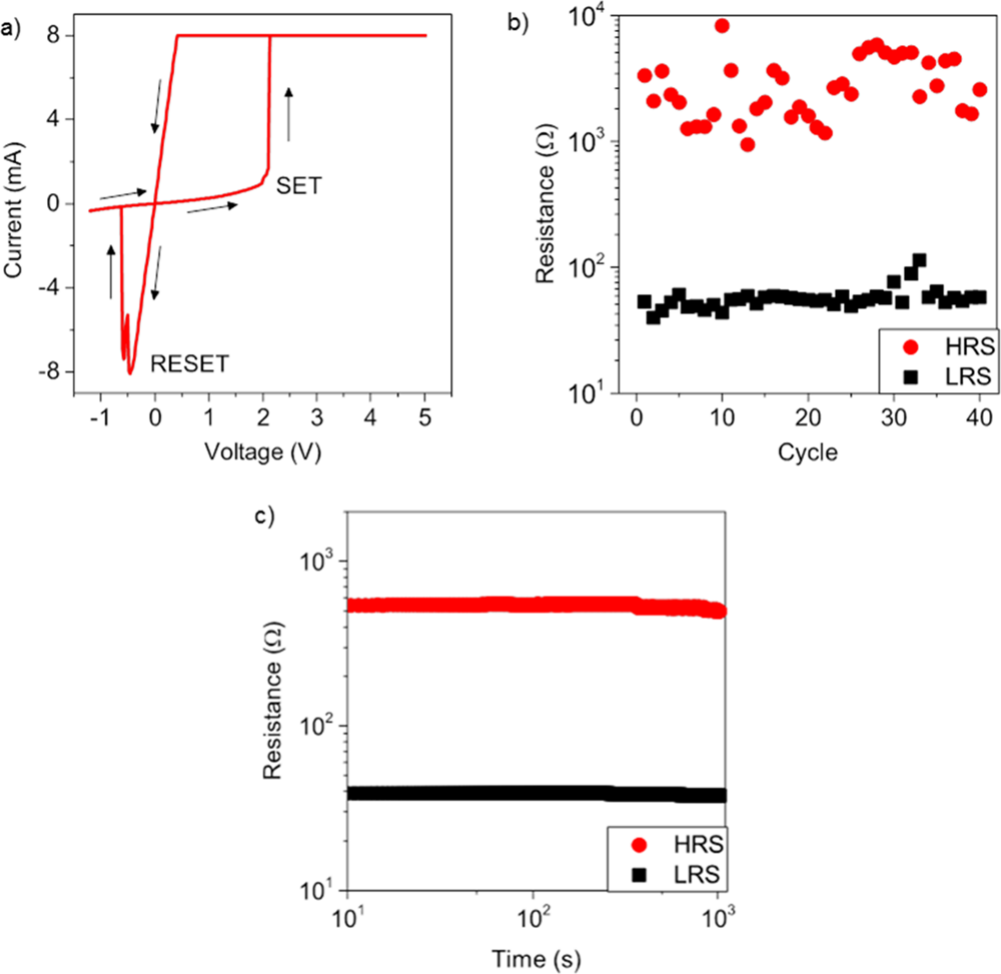
(a) Typical *I–V* cycle of a device with a CC of 8 mA (FIB dose 3), (b) endurance characteristic for the same device, acquired after some cycles of stabilization, using a reading voltage of 0.1 V, a CC of 8 mA, and (c) retention characteristic acquired using a stress voltage of 0.1 V after programming the cell with a CC of 5 mA (FIB dose 3).

After the electroforming process, the devices with FIB perturbation exhibited bipolar-resistive switching behavior, as reported in Figure 3a, where the device was operated in the voltage sweep mode. The application of a positive voltage sweep from 0 to 5 V to the Ag top electrode resulted in an abrupt change of the device resistance, turning the device from an HRS to an LRS in correspondence of the SET process occurring at the SET voltage of about 2 V. To prevent the breakdown of the cell due to Joule overheating, a compliance current (CC) of 8 mA was externally imposed to limit the maximum current flowing into the device. In the following voltage sweep from 5 to 0 V, the device remained in an LRS. Then, a negative voltage sweep from 0 to −1.2 V resulted in a RESET process where the device turned from the LRS to the HRS in correspondence of the RESET voltage of about −0.5 V. This switching mechanism is attributable to the formation/rupture of the Ag-conductive path. It should be noticed that the presence of a metallic path in the LRS is corroborated also by the Ohmic behavior observed in the LRS. The bipolar-resistive switching was observed to be reproducible over cycling, as reported in Figure 3b, where LRS and HRS values over cycling at a reading voltage of 0.1 V are reported. The mean HRS/LRS ratio was observed to be >50. Moreover, both LRS and HRS were observed to be stable over time and are reported in Figure 3c. The results of the electrical measurements performed on the devices with FIB perturbation prove that they can be used as a representative platform for material investigation.

### Observation and Understanding of Filaments

**Figure 4:**
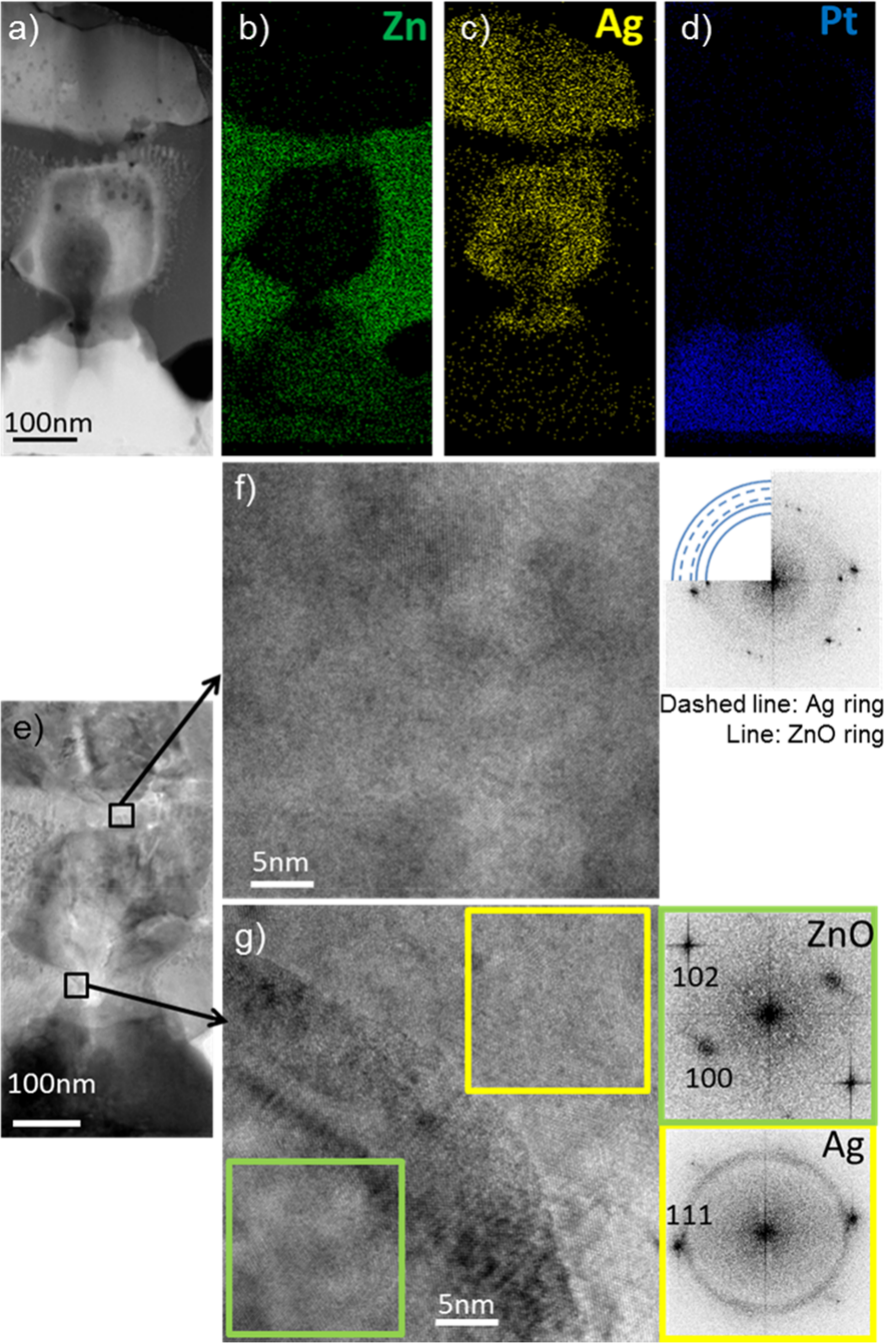
Typical Z-contrast image and EDX analysis in STEM mode for the filament region of the cycled device: (a) cross-sectional STEM image of the device, EDX maps of (b) Zn, (c) Ag, and (d) Pt; (e) BFTEM image of the same area, and HRTEM images characterizing the upper (f) and bottom (g) part of the filament, together with their FFT analysis.

The high localization of the filament formation due to the presence of the FIB-induced indentations facilitated the investigation of the morphology and structure of the filaments formed during the device testing, by means of electron microscopy and spectroscopy techniques (TEM, STEM, and EDX). The samples were processed by standard lamella FIB preparation after the electrical measurements. Figure 4a–d shows a typical cross-sectional STEM image of the bridge-like region of the cycled device and the EDX mapping results for this representative area. The top and bottom electrodes are visible, together with the ZnO switching matrix and the filament with a lateral dimension of about 200 nm. The lateral size of the observed filament is in line with other works where filaments of tens of nanometers were observed.^8,33^ The bridge appears bright in the STEM mode, similar to the Ag TE and Pt BE, which indicates that it is composed of elements with high atomic numbers. Furthermore, the EDX analyses performed in the STEM mode, showing the elemental maps of Zn, Ag, and Pt, give confirmation that the filament is composed of Ag and passes through the ZnO matrix. The EDX Ag signal is present until the Pt BE is reached. It is important to consider that the forming process induces changes to both top electrode and underlying layers, which often results in the formation of cracks within the top electrode and pores within the active layer.^40^ As a result of material reorganization, in the formed and cycled devices, there is not a clear evidence of the initially created perturbation.

EDX was also performed locally as a spot measurement in and out of the filament and is reported in Figure S5. The EDX spectrum collected out of the filament (Figure S5a) shows the contribution of Zn and O, confirming a pure ZnO (Fe, Co, and Cu derive from the TEM setup and grid). In contrast, the EDX spectrum taken from the body of the filament (Figure S5b) exhibits a very strong Ag peak, Pt that is deriving from the BE, and very low signals of Zn and O, together with a weak signal deriving from the measurement setup, as in the previous case. This difference implies that the filament might be formed via Ag percolation in the ZnO matrix, according to the ECM mechanism of switching. This however does not give any information about the crystallinity of the filament. At the first sight, it seems that a gap appears between the top electrode and bridge in correspondence of the perturbation where low-Ag and high-Zn signals are reported by EDX maps. This aspect was further investigated by HRTEM as discussed in the following. To investigate in detail the nature of the filament and matrix in its proximity, TEM was performed and is shown in Figure 4e–g. The bright-field TEM (BFTEM) image (Figure 4 e) gives an overview of the filament. In the same image, the areas inside the black squares were successively explored by high-resolution TEM (HRTEM). Figure 4g shows the HRTEM image of the interface between the filament and ZnO matrix, in a region at the end of the filament toward the BE. In this area, both the Ag filament and ZnO matrix are crystalline. FFT was performed to study the crystalline structure of both regions (the investigated areas of Ag and ZnO are highlighted by yellow and green frames, respectively). The obtained patterns confirm that the filament is largely single crystal Ag, and also, the ZnO matrix is monocrystalline.

The HRTEM image of the filament region at the opposite interface, close to the TE, is shown in Figure 4f, together with the FFT of the whole area shown. There is an evidence of higher disorder in the ZnO matrix than in the case analyzed previously. As evidenced by FFT, this area presents evidence of polycrystalline Ag and ZnO. Thus, the high-resolution images revealed that the filament is continuous and there is not a gap, but rather, the selected area of the filament has a different morphology. Therefore, it can be concluded that the filament is composed by Ag that is monocrystalline close to the BE and polycrystalline close to the TE, and the surrounding ZnO matrix tends to follow a similar behavior.

These experimental observations can be further discussed taking in consideration the ECM mechanism of filament formation, as follows: The as-prepared device is found in a high-resistance state. To set it into the ON-state, a positive voltage is applied to the active electrode (here, the Ag TE). During the set process, Ag at the interface between TE and ZnO is oxidized to Ag^+^ ions, which drift toward the Pt counter BE. There, an electrochemical reduction and electrocrystallization of Ag^+^ at the surface of the Pt electrode occur. Similarly, Ag^+^ ionic dynamics were observed also in planar devices.^15,41^ Due to this process and based on the experimental observation, we suppose that an Ag filament grows from the Pt toward the Ag electrode until an electrical contact is established, which defines the ON-state. In ECM cells, the growth of the conductive filament is regulated by kinetic parameters such as the ionic mobility that determines not only the filament shape but also the growth direction. In our case, according to Yang et al., the growth of the filament from the inert to the active electrode testifies the high mobility of Ag^+^ species in the ZnO matrix.^26^

During the device setting, heating by a Joule effect can locally lead to very high temperatures, as high as 800–900 K.^39,40^ When high temperatures are reached, a reorganization of the material structure occurs and monocrystalline filaments can be created. To reset the device back to the OFF-state, an opposite voltage is applied, which leads to a dissolution of the conducting filament. In this region, close to the TE, as observed by the measurements previously described, both Ag and ZnO remain polycrystalline. It is therefore presumable that the conduction path exists and brakes in this region during SET/RESET processes. Moreover, it is worth noticing that thanks to indentation we have observed the conductive filament in real-stacked devices differently from other works where ad hoc experiments involving planar devices were exploited for TEM investigation.^21,22^ Despite ad hoc experiments involving planar devices play a crucial role to investigate the physical phenomena underlaying resistive switching in real time,^21,22^ the planar device architecture can be not representative of real devices since surface effects are responsible for a deviation of kinetic parameters such as ionic mobility and redox rates.

**Figure 5:**
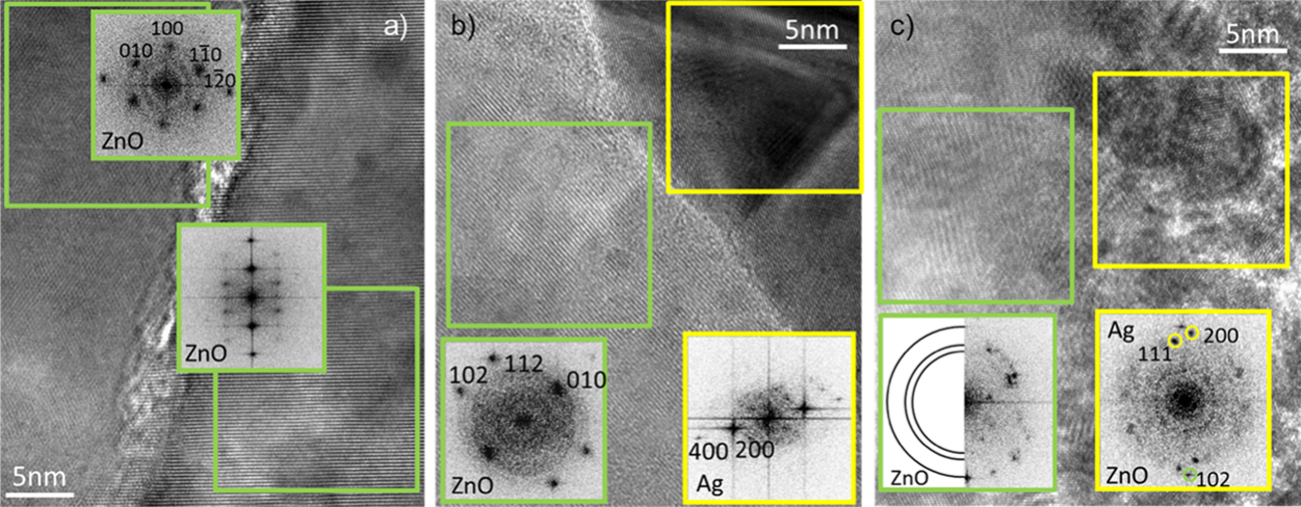
TEM images evidencing the ZnO structure of a cycled device and the corresponding FFT obtained from the area highlighted within the frame marked with the corresponding color: (a) far from the filament, (b) close to the large monocrystalline filament, and (c) close to the polycrystalline filament.

HRTEM was used to further investigate the crystallinity of both ZnO and the filament, in the samples where the bias was applied. The crystalline quality of ZnO is judged by comparing it to the as-grown material. A good crystallinity is evident in the HR-TEM by the presence of well-defined crystalline planes and in the FFT by the presence of well-defined spots. In the case of Ag, a good crystalline quality is defined by the presence of large crystals or monocrystals, in opposition to the polycrystalline material, or regions where both polycrystalline Ag and ZnO particles are present. The case shown refers to a device that was operated in voltage sweep mode for numerous cycles, after a few initial cycles were performed for stabilization (the *I–V* characterization is shown in Figure S6). The device was finally left in an ON state and the lamella was prepared for TEM characterization, and the following description is representative of the studied devices. Before cycling the device, the ZnO switching matrix shows a columnar structure composed of relatively large ZnO single-crystals with low defectiveness, separated by grain boundaries oriented perpendicularly to the electrodes, as shown in Figure S1.^9,36^ The ZnO quality in cycled devices was studied both in close proximity and away from the filament. The quality and eventual modification within the storage media were not much studied so far. The structure of ZnO far away from the filament shows good crystallinity, with two parallel crystals, as shown in Figure 5a. The FFT patterns of the two crystals show discreet points, representing a single-crystal structure for each of the grains. This shows that the original crystallinity of ZnO remains preserved in the areas of the sample where filaments are not created.

The crystallinity of ZnO close to the created metallic filaments can vary, as both monocrystalline ZnO and disordered polycrystalline ZnO were observed. Figure 5b shows an example of region where a large monocrystalline Ag filament was created, and the nearby ZnO matrix is also of good crystallinity. The FFT patterns of ZnO and Ag filament show discreet points, representing a single-crystal nature for both structures. This is very similar to the situation already shown in Figure 4g where monocrystalline ZnO and Ag were observed close to BE.

Figure 5c shows a region where the original ZnO structure has changed to a higher degree of disorder in the cycled device. The formation of the conducting filament has deteriorated the structure not only where the filament was created but also around it. This is confirmed by FFT of the ZnO grain, which does not show a signal from Ag. This result shows the polycrystalline nature of the structure, with the observation of rings from 002, 101, and 102 planes of ZnO. The part where Ag is present also shows a polycrystalline nature, mostly related to polycrystalline Ag, however, the ZnO 102 plane is present also. To some extent, the influence of Ag filaments on the crystalline structure of the ZnO matrix is expected, at least locally, as the sudden formation of a relatively large filament is likely to modify the hosting structure. Up to now, a comprehensive investigation of the crystalline structure of the filaments and matrix was not reported. However, a few works show that the forming process induces damage to both TE and the underlying layers.^40^ Schroeder et al. observed that some pores were created within the active layer,^40^ and although the mechanism was not discussed, it is reasonable to suppose a modification of the material crystalline structure. The change in the crystallinity of the matrix was also observed by Yang et al. in ZnO:Mn during the forming process^8^ where the *c*-axis oriented polycrystalline wurtzite structure changed to a more disordered polycrystalline wurtzite structure. In this case, the measurement was performed with the use of selected area electron diffraction (SAED); therefore, a quite large area was investigated simultaneously; hence, it is not clear whether all ZnO:Mn have changed, or only some areas, possibly close to the filaments in the area where the switching occurs. Our analysis confirmed that the crystalline structure of the switching material depends on the position within the device in respect to the electrodes and filament.

The above-presented main filaments are representative of the working devices. We have also tested the morphology of the filament after complete breakdown of the device, as reported in Figure S7. The failure was induced by the application of increasing values of CC for setting the device into the ON state, then switching to OFF state, and setting it again with higher CC (up to 35 mA). The *I–V* of the irreversible forming with a CC of 35 mA is shown in Figure S7a. After that, it was not possible to reset the device anymore; therefore, the device can be considered to have reached failure, remaining in the ON state. Figure S7b shows a cross-sectional STEM image of the bridge region of this device after failure. This was acquired using an HAADF detector, which is very sensitive to the atomic number, and the brightest parts of the image are composed of elements with high atomic numbers. As can be observed from Figure S7b,c, the filament morphology after device failure strongly differs from the filament shape and is reported in Figure 4a. In this device, there is in fact a strong and nonuniform connection between the top and bottom electrodes, suggesting that the ZnO switching matrix is not present any more between electrodes and that the top and bottom electrodes are connected via a large metallic bridge. The STEM and BFTEM images (Figure S7b,c) show that the bridge is made of crystals with variable dimensions. The HRTEM performed revealed that these crystals are composed of Ag, and the bridge is made of Ag that is locally monocrystalline, as shown in Figure S7d.

**Figure 6:**
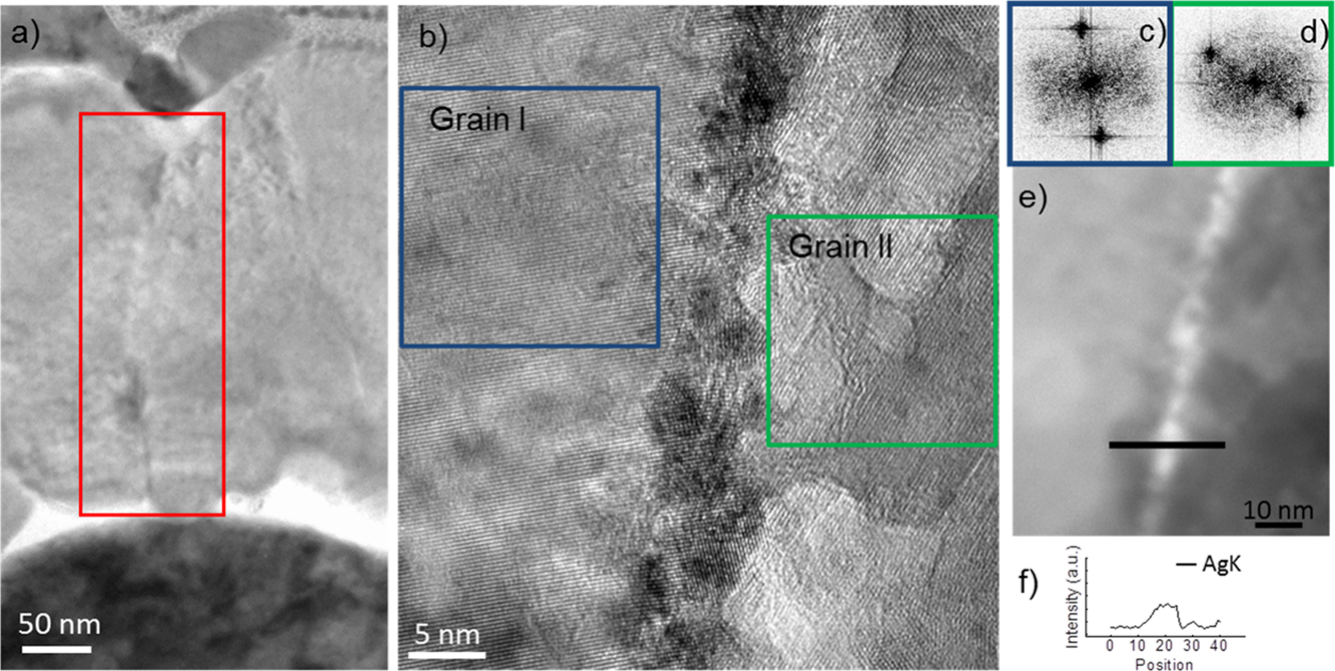
TEM characterization of a cycled device of the region far from the FIB perturbation where a narrow secondary Ag filament is created: (a) BFTEM image of a the area where Ag filament is created between two grains present, the red frame indicates the area where the filament was formed; (b) HRTEM image showing a narrow secondary Ag filament at the boundary between two ZnO grains (Grains I and II); (c, d) FFT of Grains I and II, confirming good quality ZnO monocrystals, with different orientation, respectively; (e) STEM image of the Ag secondary filament; and (f) EDX profile of the black line in panel e, showing the EDX intensity of Ag across the filament.

In addition to the large main filament obtained in proximity of the FIB perturbations, we have found evidence that secondary narrow filaments are likely to create also at ZnO grain boundaries. Extended defects in the active material layer, including grain boundaries, are considered to facilitate the filament formation, although so far, the filament formation at the grain boundaries was not observed in the case of ECM. These filaments were observed at the distance of a few hundreds of nanometers from the main filaments. Figure 6a reports a BFTEM image of a cycled device where two ZnO grains are visible, and in between them, a narrow secondary Ag filament is present; however, it cannot be seen at such low magnification. The HRTEM image in Figure 6b shows a grain boundary between two ZnO crystal grains where it is possible to observe a secondary Ag filament. The FFT patterns shown in Figure 6c,d were obtained from grains at both sides of the small filament, from the regions highlighted by frames with corresponding color. They show that both grains are composed of good quality monocrystalline ZnO, with different orientation. It is difficult to study the filament on the basis of the HRTEM image only, as it is formed of many small overlapping crystals. Figure 6e shows the STEM image of the filament in between two grains. We observe that the narrow filament is bright, which indicates that it is composed of elements with high atomic number. In Figure 6f, an EDX line profile of the structure observed in Figure 6e shows signal from Ag at the filament region, without the presence of Ag signal in the surrounding grains, confirming that the crystalline structure observed at the grain boundary is polycrystalline Ag.

**Figure 7:**
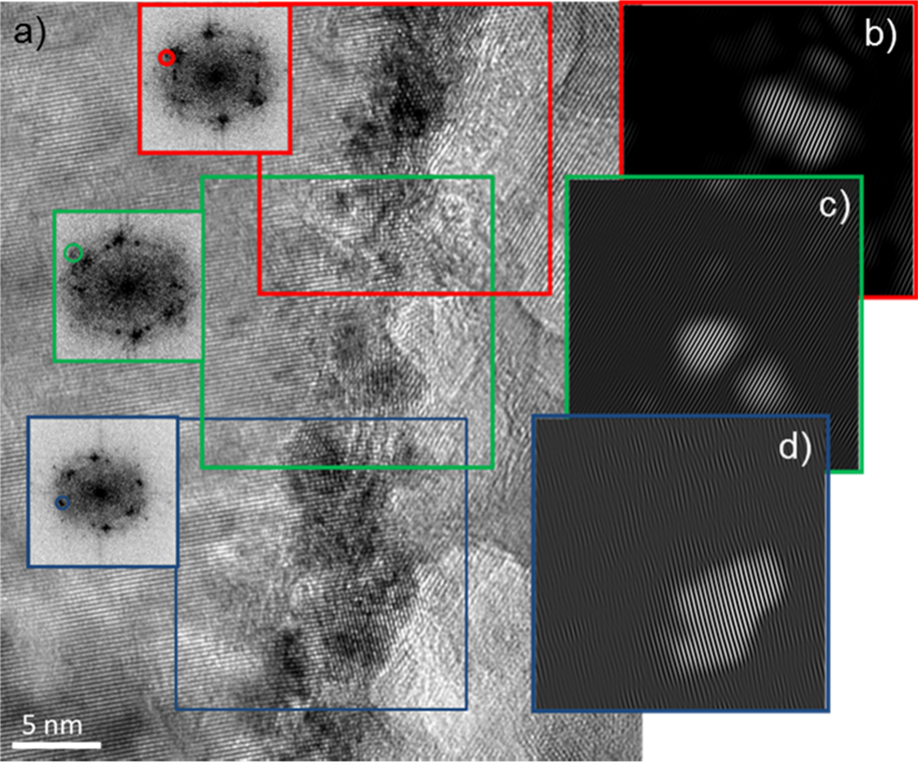
(a) HRTEM image of a region far from the FIB perturbation in a cycled device (the same image of Figure 6b), and the corresponding FFT obtained from the area highlighted within the frame marked with the corresponding color. (b)–(d) Digital dark field decomposition obtained by means of selective masking and filtering of the FFT (where only one couple of spots was selected). From these images, it is possible to observe the filament and its dimensionality in more detail, as the crystals in the filament are well evidenced.

To decouple the signal of Ag from ZnO, and obtain more information about the Ag secondary filament, the FFT patterns were derived from the HRTEM image shown in Figure 6b, and are shown in Figure 7.

The FFT patterns in Figure 7a were taken in several points along the filament, from the regions highlighted by frames with corresponding color. The diffraction spots visible in FFT patterns derive from both filament and ZnO. Digital dark field decomposition is obtained by means of selective masking (only one couple of spots, related to metallic Ag, was selected, as shown) and filtering of the FFT. The inverse FFT patterns obtained by selective masking (Figure 7b–d) show Ag crystals with the lattice spacing and orientation corresponding to the selected diffraction spots. Using this approach, it is possible to obtain information about the crystals composing the filament and estimate their sizes. This analysis shows that the Ag filament is polycrystalline and made of nanoparticles with a size range of about 4–10 nm, which distribute at the ZnO grain boundary. These types of secondary filaments were formed at some grain boundaries, in addition to the large filament created at the perturbation.

The structure and morphology of the observed filaments discussed above are representative of devices studied. Although large differences of forming voltage in the function of the protrusion depth were observed, the structural properties after forming were comparable. In addition, after forming, it is actually difficult to see any difference between the devices in the function of the dose applied, therefore of the perturbation depth.

To the best of our knowledge, this is the first work reporting that the metallic filament (Ag) created in resistive switching devices is in large part monocrystalline, although sporadic polycrystalline Ag zones were also found. As already observed in other works where Ag filament was created in ZnO-based matrix via the ECM mechanism, the quality of ZnO matrix degrades during the cycling.^8^ However, we have observed that the crystalline quality of the matrix can be quickly recovered because ZnO with good crystallinity can be observed around the large main switching filament, remaining polycrystalline mainly only at the switching area. It is reasonable that structural changes are happening during each set/reset, and that this is locally limited to the polycrystalline switching zone. However, further experiments are needed to elucidate the degradation/recovering of the quality of the ZnO matrix and Ag over cycling.

The change in the crystalline structure can result from the fact that very high temperatures can be reached within the memristor during the application of the electrical bias; in particular, they are expected to raise locally at the sites where conductive filaments are formed.^42,43^ In addition, it can be speculated that it is indeed the local high temperature that leads to structure reorganization and creation of the single-crystal structure, starting from the initial polycrystalline Ag. The melting point of bulk Ag is about 962 °C; thus, it is expected to be much lower for Ag nanoparticles. For 5 nm-sized nanoparticles, it can be as low as 530 °C.^44^ This effect is ascribable to a much weaker surface bounding in nanoparticles, as they do not possess full coordination; therefore, the surface can melt quicker than the bulk. In the present case, it is important to consider also that Ag nanoparticles are surrounded by the ZnO matrix, which at this stage of the switching process does not possess a high crystalline quality, too. Therefore, the filament and surrounding area, which are made of nanoparticles, have a greater surface area per unit volume compared with the bulk materials and the melting temperature is decreased. Although a fine approximation of the real melting temperature of the nanoparticles composing the observed filaments and surrounding area is difficult to predict, relatively high temperatures can be reached, as already observed in other works about memristor devices,^42,43^ and can be responsible for a reorganization of the crystals with the formation of a monocrystalline structure.

## Summary and Conclusions

This work investigates the nanostructure of Ag-conductive filaments in resistive switching devices based on polycrystalline ZnO thin layers grown by LPCVD, sandwiched between Ag non-inert and Pt inert electrodes in which a perturbation was introduced by FIB-assisted technology to promote the formation of localized filaments. The described procedure allows direct observation of the switching filaments, by the preparation of lamellas at the precise filament location for subsequent high-resolution characterization by transmission electron microscopy and spectroscopy. A comprehensive structural characterization of the switching filaments and surrounding storage media is thus achieved by HRTEM and STEM, which results in *Z*-contrast imaging, as well as EDX analysis. This study enriches the understanding of the material structure of both the active region and conducting filament, giving information about the structural modification that they go through in memristive devices.

Our work revealed the existence of multiple filaments since, in addition to large filaments created at the perturbation, smaller filaments were also created at the grain boundaries between ZnO crystals in the switching matrix. In all cases, the filaments are mainly composed of metallic Ag, which shows characteristics of monocrystallinity close to the bottom inert electrode, and polycrystallinity close to the top active electrode and throughout the whole secondary filaments found at the grain boundaries. Assuming that the decrease or increase of resistivity while switching ON and OFF the device occurs mainly at the primary filament induced at the artificial perturbation, it is possible to infer that the rupture of the filament occurs close to the top active electrode where polycrystalline Ag is observed, while monocrystalline Ag close to the bottom inert electrode remains unchanged. Also, we observed that the polycrystalline ZnO matrix in the switching areas can change its crystallinity to a higher degree of disorder. Finally, the disordered polycrystalline structure of the filaments and surrounding ZnO matrix, which is created during the application of the electrical bias to the device, is largely recovered quickly afterward, leading to monocrystalline structure of both the filament and ZnO matrix, which are found along most of the filament. We suggest that this effect takes place as a result of a localized increase of temperature.

## Supporting Information Available

TEM characterization of the as-grown polycrystalline film in OFF-state (S1), FESEM image showing multiple devices created (S2), Pristine state of the Ag/ZnO/Pt device showing diode-like behavior (S3), additional *I–V* characteristics: pristine state and electroforming of the devices with perturbations formed by means of different FIB doses (S4), EDX characterization performed locally in the area surrounding the filament and at the filament (S5), electrical *I–V* characterization of the device shown in Figure 5 (S6), characterization of the device after failure induced by the application of increasing values of compliance current for setting the device into the ON state (S7) (PDF)
