# Supplementary material for: TEM Nanostructural Investigation of Ag-Conductive Filaments in Polycrystalline ZnO-Based Resistive Switching Devices
Source: ACS Appl Mater Interfaces. 2020 Jun 8;12(26):29451–60. doi: 10.1021/acsami.0c05038 (PMC8008384; doi:10.1021/acsami.0c05038)
Supplement: Supplementary file 1 — am0c05038_si_001.pdf [file am0c05038_si_001.pdf]

Supporting Information

TEM nano-structural investigation of Ag conductive filaments in polycrystalline ZnO-based resistive switching devices

Katarzyna Bejtka[†*], *Gianluca Milano*[†§], *Carlo Ricciardi*[§], *Candido F. Pirri*[†§] *and Samuele Porro*[§].

[†]Center for Sustainable Future Technologies @ POLITO, Istituto Italiano di Tecnologia, Via Livorno 60, 10144 Turin, Italy.

[§]Department of Applied Science and Technology, Politecnico di Torino, C.so Duca degli Abruzzi 24, 10129 Torino, Italy.

Katarzyna Bejtka: katarzyna.bejtka[at]iit.it;

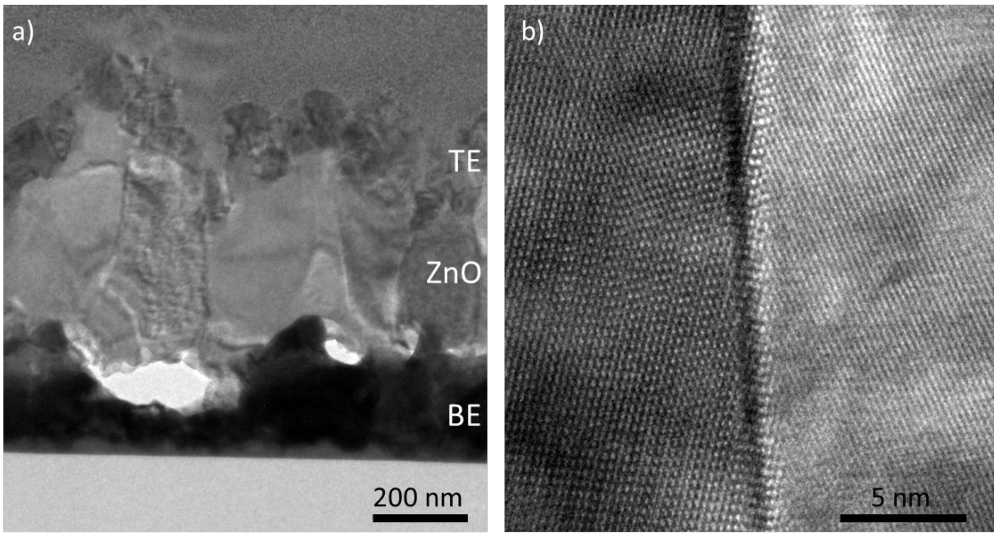

Figure S1. Cross section TEM image of the as-grown polycrystalline film in OFF-state (a) Bright field TEM showing ZnO sandwiched between top (TE) and bottom (BE) electrodes, (b) HRTEM image of the interface between two grains of ZnO. These images show the as-grown material without introduction of the perturbation for easy comparison of the material quality.

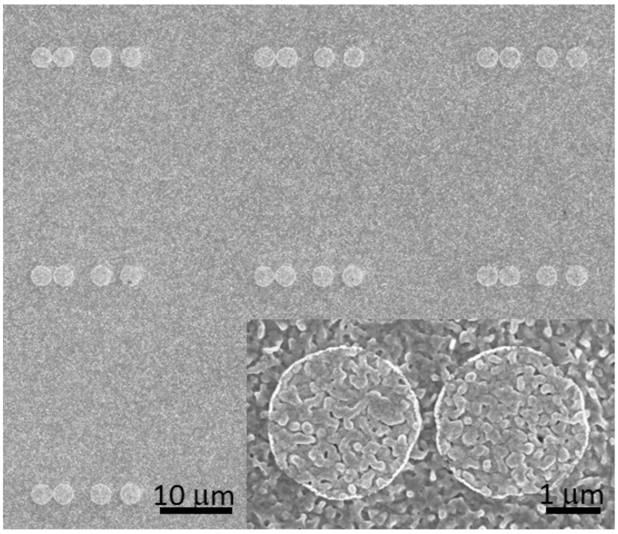

Figure S2. Top view FESEM image showing multiple devices created.

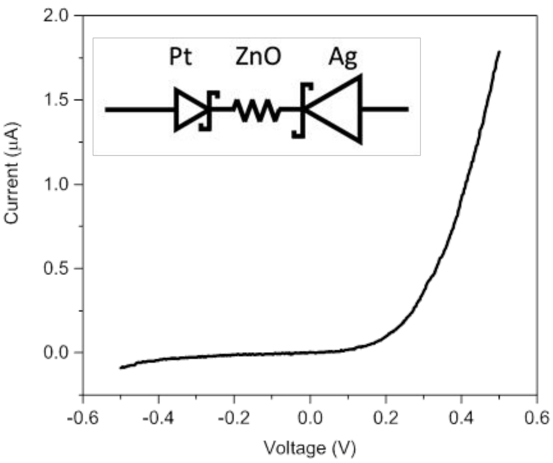

Figure S3. Pristine state of the Ag/ZnO/Pt device exhibiting diode-like behaviour attributable to the Schottky barriers at the metal/semiconductor interface, as discussed in previous works. (sample dose 1). The inset shows the electrical schematization of the Ag/ZnO/Pt device that can be schematized as a back-to-back Schottky diode. The asymmetric curve results from the asymmetric junction properties due to the different metal work function and different chemical properties at the metal/semiconductor interface.

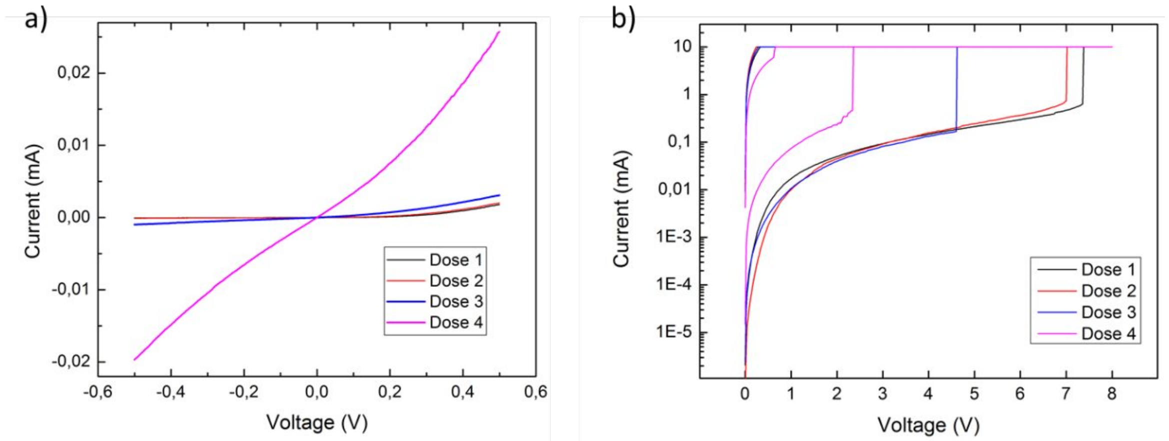

Figure S4. (a) Pristine state and (b) electroforming of the Ag/ZnO/Pt devices with perturbations formed by means of different FIB doses, where dose 4 > dose 3 > dose 2 > dose 1, as detailed in the experimental section.

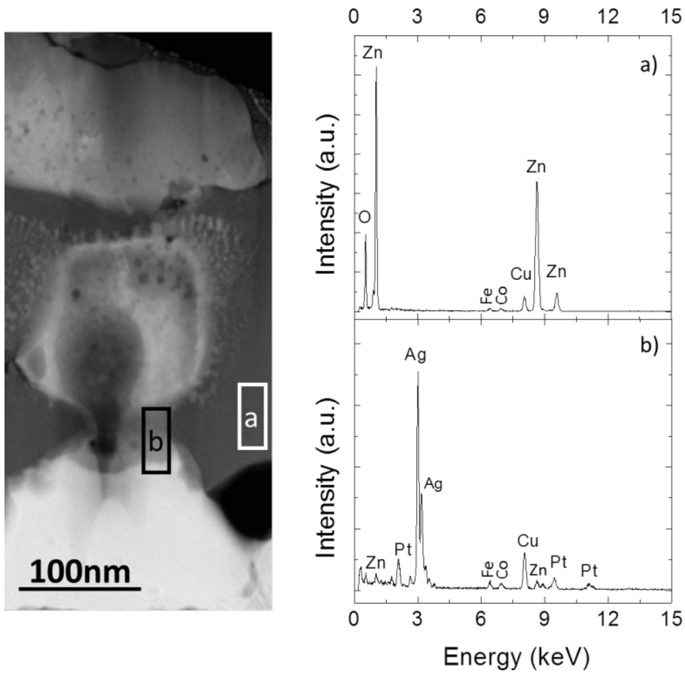

Figure S5. EDX spectra performed locally (a) area surrounding the filament; (b) filament.

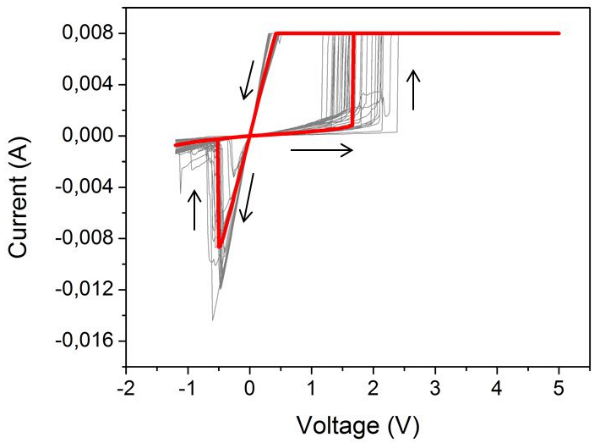

Figure S6. Electrical I-V characterization of the device shown in Figure 5. Grey curves show the collected data for 40 cycles performed after stabilization of the device; the red curve is highlighted for ease of viewing.

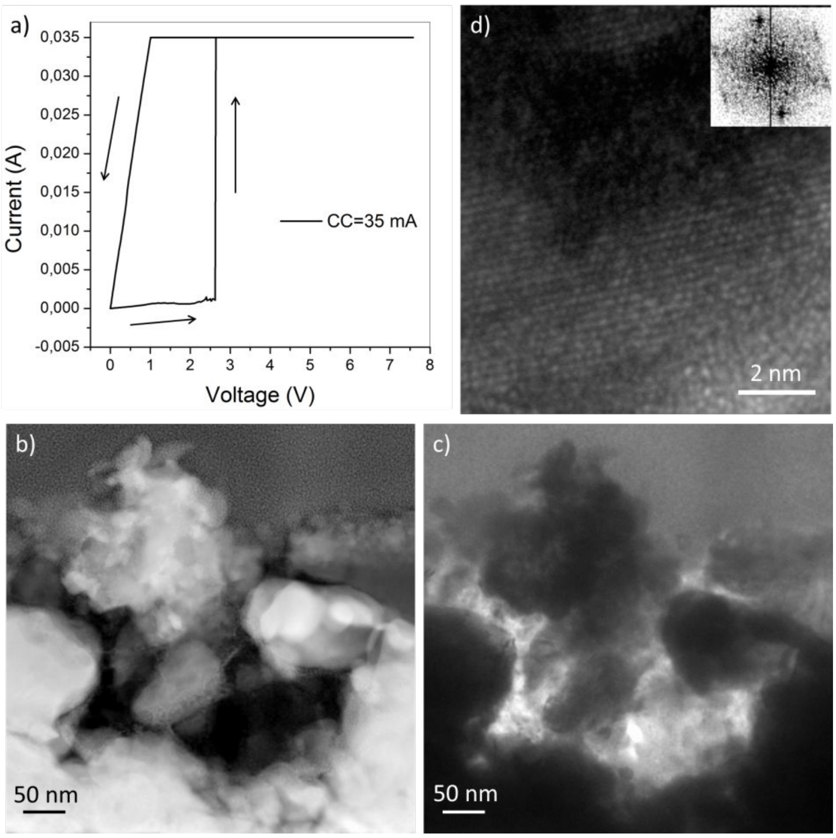

Figure S7. Characterization of the device after failure induced by the application of increasing values of compliance current for setting the device into ON state, then switching to OFF state, and setting it again with higher CC (up to 35 mA): (a) I-V cycle of the device formed with CC of 35 mA; TEM characterization of the filament region of the cycled device after failure: (b) HAADF STEM image, (c) BF-TEM image of the same area, (d) HR-TEM image with the FET in the inset.
